# Vitamin and Mineral Supplement Use among Korean Adults: Baseline Data from the Trace Element Study of Korean Adults in Yeungnam Area

**DOI:** 10.3390/nu10010050

**Published:** 2018-01-06

**Authors:** Minkyeong Kim, Yujin Lee, Kyong Park

**Affiliations:** Department of Food and Nutrition, Yeungnam University, Gyeongsan, Gyeongbuk 38541, Korea; minkyeong@ynu.ac.kr (M.K.); yj_lee@yu.ac.kr (Y.L.)

**Keywords:** dietary supplements, vitamins, minerals, Koreans, nutrient intake, recommended dietary intake

## Abstract

Although dietary supplement use is believed to improve health status, the efficacy and safety of its use remains controversial. This study aimed to investigate the contribution of consumption of vitamin and mineral supplements (VMS) to the total micronutrient intake. Study participants (*n* = 586) were selected from the ongoing prospective cohort study of the Korean population, and baseline information on current use of dietary supplements, types of supplements, frequency of use, dosage, duration, and brand name was collected. Dietary information was assessed using a 146-item semi-quantitative food frequency questionnaire. Approximately one-fourth of the participants were categorized as VMS users. The proportion of VMS use was significantly higher in women (*p* = 0.02), older participants (*p* = 0.002), and those with a higher income level (*p* = 0.03) than in non-users. All vitamin and mineral intakes of both groups met the recommended nutrient intake levels by food consumption alone, except for riboflavin, calcium, and magnesium. Approximately 0.7–3.4% of the VMS users had nutrient intake levels that exceeded the tolerable upper intake levels for vitamin A, E, C, iron, and iodine. Excessive use of VMS can lead to an increased risk for adverse health effects. The results of this study provide useful baseline data for establishing guidelines for the appropriate consumption and adequate intake levels of VMS.

## 1. Introduction

Today, individuals are becoming more health conscious and are searching for better ways to prevent chronic diseases. Individuals who take dietary supplements reportedly believe that dietary supplements can assist in maintaining health, prolonging lifespan, and promoting recovery from fatigue [1,2,3].

According to the results of the 2015 Korean National Health and Nutrition Examination Survey (KNHANES), approximately 42% of Korean adults aged 19 years or older have taken a dietary supplement at least once in their lifetime [4]. The global dietary supplement market, including that in Korea, has been consistently expanding, and growing demands for dietary supplements have diversified the types of dietary supplements available [5,6].

While dietary supplement use is believed to be beneficial for improving health status and preventing chronic diseases [2,7], the efficacy and safety of dietary supplement use remains controversial. One of the beneficial effects of dietary supplements is that it can contribute to achieving the recommended nutrient intake (RNI) for some micronutrients in certain populations [8,9]. Furthermore, dietary supplements can serve as treatment for serious nutrient deficiencies or for the prevention of some diseases [10]. Iron supplements for anemia [11], folic acid supplements for neural tube defects during pregnancy [12], niacin supplements for hyperlipidemia [13], and magnesium supplements for improving insulin sensitivity [14] are some of the prominent examples. In contrast, one concern is that an excessive intake of nutrients from dietary supplements can cause adverse health effects [15]. A recent meta-analysis of randomized trials investigating the association between calcium supplement intake and cardiovascular disease reported that at least 500 mg/day of calcium supplementation caused an approximately 27% increase in the risk for myocardial infarction [16]. In the cohort of Swedish men, excessive vitamin C and vitamin E intakes (1000 mg and 100 mg, respectively) were shown to significantly increase age-related cataract incidences [17], although these excessive intake levels were lower than the tolerable upper intake level (UL). Another concern regarding dietary supplement use is that even when the amounts of nutrients consumed from foods and dietary supplements are equivalent, their efficacies and health effects in the body may vary. For instance, in a study comparing the effects of tomatoes and refined lycopene supplements on the incidence of prostate cancer, tomatoes were more effective than lycopene supplements in inhibiting the onset of prostate cancer [18]. In addition, Michos et al. reported that higher calcium intake was associated with a decreased risk of incident atherosclerosis, whereas calcium supplement intake was associated with an increased risk of incident atherosclerosis [19]. Furthermore, the bioavailability and health effects of nutrients vary depending on their chemical form and sources, particularly for niacin, magnesium, and folate/folic acid [20,21,22,23].

To date, although the proportion of Korean adults using dietary supplements is as high as 43% [4,24,25], only a few studies have examined the contributions of vitamin and mineral supplements (VMS) to the overall micronutrient intake of Korean adults in comparison to the Korean dietary reference intakes. Therefore, this study aimed to investigate the contribution of nutrient intakes from food and VMS to the total micronutrient intake among individuals who use VMS and those who do not use VMS.

## 2. Materials and Methods

### 2.1. Study Population

A total of 740 adults were recruited at baseline for the Trace Element Study of Korean Adults in Yeungnam Area (SELEN), which is an ongoing prospective cohort study of healthy middle-aged Korean adults residing in the Yeungnam area of Korea. The details of the SELEN cohort study have been previously described [26,27,28,29]. Out of the 740 adults, 618 responded to the dietary questionnaire. The age of the participants ranged from 28 to 66 years. The exclusion criteria were as follows: (1) a reported implausible total energy intake ( < 500 or >5000 kcal/day, *n* = 1) [30], (2) no information on dietary supplement use (*n* = 18), (3) pregnant or lactating women (*n* = 1), (4) chronic disease (*n* = 9) such as cardiovascular disease or cancer, and (5) receiving treatment for iron deficiency (*n* = 3). In total, 586 participants were included in the final analysis. This study was approved by the Institutional Review Board (IRB) at Yeungnam University Medical Center, and informed consent was obtained from all participants before collecting the data (IRB: YUH-12-0468-O94).

### 2.2. General Characteristics

Participants’ sex, age, monthly household income level, education level, smoking status, alcohol drinking status, body mass index (BMI), family history of disease, and dietary information were collected from each participant using a self-reported questionnaire. Age was calculated by subtracting the participant’s birthdate from the date of the questionnaire, and monthly household income level was classified into three groups (<3 million Korean Republic Won (KRW), 3–<5 million KRW, ≥5 million KRW). Education level was divided into two categories: below high school graduation and above college graduation. Alcohol drinking status was assessed by inquiring about the usual frequency of alcohol consumption over the past year. Smoking status was categorized into non-smoker, former smoker, and current smoker. BMI was calculated using the weight and height information recorded in the self-reported questionnaire, with weight in kilograms divided by the square of height in meters. The calculated BMI was classified into underweight/normal (<23 kg/m^2^), overweight (23–<25 kg/m^2^), and obese (≥25 kg/m^2^), based on BMI criteria from the World Health Organization for Asian populations [31].

### 2.3. Dietary Information

Dietary intake was assessed using the validated 146-item semi-quantitative food frequency questionnaire (SQFFQ) that was developed in consideration of dietary characteristics specific to the SELEN participants [27]. The participants were instructed to choose the frequency and amount of each food item consumed over the past year. The ten categories of frequency were as follows: rarely, fewer than once per month, once per month, two to three times per month, once per week, two to four times per week, five to six times per week, once per day, two times per day, and more than three times per day. The portion size of each food item consumed was reported as small (0.5 times the reference), medium (reference) and large (1.5–2 times the reference).

Data on the current use of dietary supplements were obtained from a subset of the dietary questionnaire. For those who responded affirmatively, the following additional information was sought: type of dietary supplement taken, weekly frequency of use, daily dosage, duration of use, and brand name of the product. For this study, VMS supplement users were defined as those consuming at least one type of VMS (single vitamin, mineral, or multivitamins/minerals) per week. Health products, functional foods, or herbal products in which information on vitamin or mineral content was not available were excluded. Daily nutrient intake levels from VMS were calculated in terms of the dosage and frequency of intake per day, as well as product-specific formulations. To calculate and compare nutrient intakes from foods and VMS, some nutrients were converted into standard units using a conversion coefficient [32].

Nutrient intakes were compared with dietary reference values [33]. In order to evaluate whether the participants had sufficient intake levels to meet the daily nutrient requirements, percentages of RNI were calculated for the following nutrients: vitamin A, vitamin C, thiamin, riboflavin, niacin, vitamin B_6_, folate/folic acid, vitamin B_12_, calcium, phosphorus, magnesium, iron, zinc, copper, iodine, and selenium. In addition, the percentages of intake levels above the UL for vitamin A, vitamin D, vitamin E, vitamin C, niacin, vitamin B_6_, folic acid, calcium, phosphorus, iron, magnesium, zinc, copper, fluoride, manganese, iodine, and selenium were calculated. The CAN Pro version 4.0 (the Korean Nutrition Society, Seoul, Korea) and the Korean Food Composition Table version 9.0 [34] were used to assess nutrient intake levels.

### 2.4. Statistical Analysis

The general characteristics of the participants were compared between VMS users and non-users. The chi-square test and a general linear regression analysis were used for categorical and continuous variables, respectively. All statistical analyses were performed with SAS version 9.4 software (SAS Institute Inc., Cary, NC, USA), and statistical significance was set at *p* < 0.05.

## 3. Results

The general characteristics of the participants in relation to their use of VMS are presented in Table 1. Approximately 25.4% of the study participants were categorized as VMS users, and the proportion of VMS use was significantly higher in women (*p* = 0.02), older participants (*p* = 0.002), and those with a high monthly household income level (*p* = 0.03), when compared with non-users. However, there were no significant differences in education level (*p* = 0.2), smoking status (*p* = 0.1), alcohol consumption (*p* = 0.5), BMI (*p* = 0.8), medical condition (*p* > 0.1), or family history of disease (*p* = 0.1) between supplement users and non-users.

**Figure 1:**
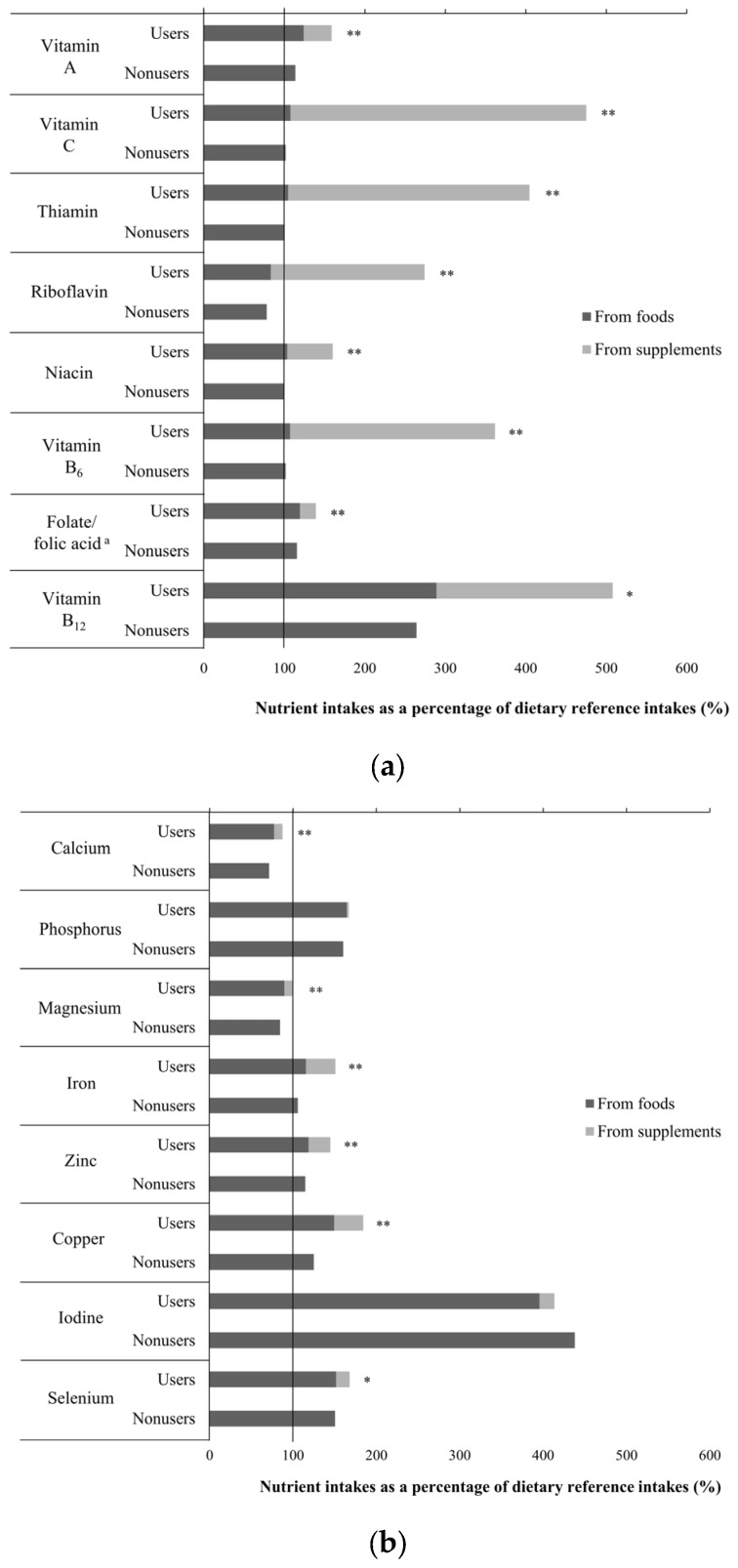
(**a**) Vitamin intakes as a percentage of dietary reference intakes (*n* = 586). The solid vertical line indicates the Korean dietary reference intakes. The black bar indicates intake levels from foods only. The gray bar indicates the intakes from vitamin and mineral supplements (VMS). *p* values are derived from the generalized linear regression analysis for the difference in nutrient intakes when compared to recommended nutrient intakes between VMS users and non-users. * *p* < 0.01, ** *p* < 0.001. ^a^ The black bar indicates folate intake levels from food sources and the gray bar indicates folic acid intake levels from supplements. (**b**) Mineral intakes as a percentage of dietary reference intakes (*n* = 586). The solid vertical line indicates the Korean dietary reference intakes. The black bar indicates intake levels from foods only. The gray bar indicates the intakes from VMS. *p* values are derived from the generalized linear regression analysis for the difference in nutrient intakes when compared to recommended nutrient intakes between VMS users and non-users. * *p* < 0.01, ** *p* < 0.001.

A comparison of intake levels of vitamins and minerals from foods and supplements between VMS users and non-users is shown in Figure 1. Overall, VMS users had a substantially higher level of vitamin intake, as compared to non-users, especially for vitamin A (*p* < 0.001), vitamin C (*p* < 0.001), thiamin (*p* < 0.001), riboflavin (*p* < 0.001), niacin (*p* < 0.001), vitamin B_6_ (*p* < 0.001), folate/folic acid (*p* < 0.001), and vitamin B_12_ (*p* < 0.01, Figure 1). Except for riboflavin, the intake levels of all vitamins from food alone met or exceeded the RNI for both users and non-users. Importantly, the intake of vitamin B_12_ exceeded the RNI by 289% and 264% for VMS users and non-users, respectively. Furthermore, VMS users tended to consume an excessive amount of vitamin B_12_ through additional supplements in comparison to the RNI (508%); this was also observed for vitamin C (475%) and thiamin (405%). In addition, the mineral intakes in most VMS users and non-users met the RNI, except for calcium and magnesium (Figure 1). Even VMS users demonstrated calcium intake levels below the RNI. In contrast, the intake of iodine from foods alone exceeded the RNI dramatically (396% and 438% in users and non-users, respectively).

**Figure 2:**
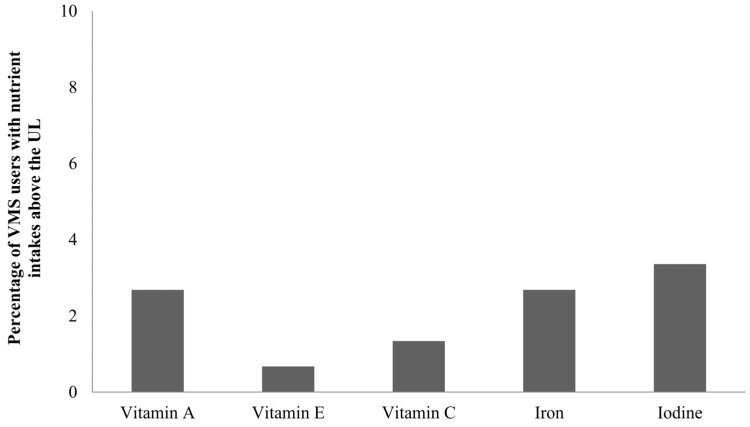
Percentage of vitamin and mineral supplements (VMS) users receiving nutrients from food and VMS above the tolerable upper intake level (UL).

The intake levels of some nutrients, such as vitamin A, vitamin E, vitamin C, iron, and iodine exceeded the UL among VMS users. Figure 2 shows the percentage of VMS users whose nutrient intake exceeded the UL. Among VMS users, the iodine intake most frequently exceeded the UL (3.4%), followed by vitamin A (2.7%), iron (2.7%), vitamin C (1.3%), and vitamin E (0.7%).

## 4. Discussion

In this study, the recommended intake levels for most vitamins and minerals were satisfied by consumption of food alone, and some nutrients were even consumed in excess of the recommended intake levels mainly due to dietary supplement use, except for iodine. The excessive intake of iodine may be due to the Koreans’ habitual consumption of seaweed. Further, up to 3.4% of VMS users had substantially high levels of intake that exceeded the UL, especially for vitamin A, vitamin E, vitamin C, and iron.

These results are consistent with those reported in other previous studies in Korea [35,36,37], which consisted of study populations with similar ages and demographic characteristics. Similarly, a multicultural cohort study of Hawaii and California residents found that the consumption of dietary supplements led to an excessive nutrient intake, especially folic acid and niacin [38]. In addition, approximately 4–11% of American adults in the National Health and Nutrition Examination Survey who took dietary supplements had calcium, iron, zinc, and magnesium intake levels exceeding the UL [39].

Excessive nutrient intake from VMS may lead to adverse health effects. A previously published meta-analysis showed that supplement intakes of beta-carotene (1.2–50 mg), vitamin A (1333–200,000 IU), and vitamin E (10–5000 IU) were associated with elevated mortality rates [40]. In addition, studies have indicated that purified nutrients may not always have the same biological effects in different sub-groups. For example, high doses of beta-carotene were found to be associated with increased lung cancer risk in heavy smokers, while this detrimental effect was not observed in healthy populations [41]. In addition, there are concerns that an excessive intake of folic acid can lead to adverse side effects, such as masked vitamin B_12_ deficiency [42], dysregulation of folate metabolism [10], and even increased incidence of cancer [43].

The bioavailability of nutrient supplements varies depending on their chemical form [21,22,23]. For instance, folic acid, a synthetic monoglutamate form present in fortified food or supplements, has a much higher bioavailability than folate and polyglutamates from natural foods [21]. The absorption of niacin from food sources is lower than that for nicotinic acid or nicotinamide, which can involve side effects [22]. In addition, several lines of evidence suggest that high doses of magnesium supplementation can cause gastrointestinal side effects, which are not observed in magnesium derived from foods with similar amounts [23].

In the present study, approximately 60% of the participants were taking dietary supplements, including herbal products and functional foods (data not shown), but VMS were the most prevalent type of dietary supplement (43%, data not shown). A previous study using KNHANES data also found that vitamin- or mineral-based products were the most frequently used dietary supplements among the Korean population [44]. Similarly, multivitamins were the most frequently used supplement type [45] among children, whereas adolescents most frequently consumed vitamin C products, followed by multivitamins and vitamin A [2]. A prior study of US residents showed that approximately 54% of American adults used dietary supplements, with multivitamins or minerals being the most popular supplement products (33%) [46].

We also found that VMS users had significantly higher intakes of all selected micronutrients than non-users, except for iodine and phosphorus. There were no major differences in mineral intake levels between users and non-users, ranging between 6.7% and 59.4% of the RNI in phosphorus and copper, respectively. In contrast, differences in vitamin intake levels were relatively larger between the two groups (60.9% and 373.3% of the RNI for niacin and vitamin C, respectively). However, it is uncertain whether these differences would have any clinical relevance. Nonetheless, even the VMS users did not meet the RNI for calcium, and barely met the RNI for magnesium. In this case, VMS use could be beneficial to achieve adequate intake levels of these nutrients.

This study had several limitations. First, a self-reported questionnaire was used to estimate the micronutrient intake levels from foods and dietary supplements, which may lead to misclassification. To account for this limitation, the investigator reviewed each questionnaire, and any omissions or unclear responses were confirmed via additional telephone interviews with the participants. Although VMS were the most frequently used form of dietary supplements, the study exclusively evaluated the nutrient intake levels of vitamin- or mineral-based products for which nutrient data are available, rather than examining all types of dietary supplements. Therefore, nutrient intake levels from dietary supplements may be underestimated. Lastly, the study participants were self-selected and limited to residents of the Yeungnam area of South Korea; therefore, the generalizability of the results of this study result may be limited.

The strength of this study is that a validated SQFFQ which considered regional and cultural features was used to identify the dietary intakes of participants. In addition, nutrient intake levels from foods and VMS were evaluated, enabling the investigation of the contribution of nutrient intake levels from separate sources to total micronutrient intakes.

## 5. Conclusions

Both VMS users and non-users met their micronutrient needs predominantly through food alone. In addition, excessive use of VMS can lead to an increased risk for adverse health effects. However, those who might require additional supplementation owing to recommendations, such as pregnant or lactating women, those with nutrient deficiencies or cancer, or those under special circumstances, should consider using VMS to meet their nutrient needs. A long-term cohort study is needed to elucidate the benefits and adverse effects of dietary supplements. The results of the present study can be useful as baseline data for establishing guidelines for appropriate use and adequate intake of dietary supplements.

## Figures and Tables

**Table 1:** General characteristics of the participants by use of vitamin and mineral supplements (VMS) ^1,2^ (*n* = 586).

	Non-Users	VMS Users	*p* Value ^3^
N	437 (74.6)	149 (25.4)	
Sex, men	219 (50.1)	58 (38.9)	0.02
Age, years	44.6 ± 5.4	46.3 ± 5.9	0.002
Monthly household income level, KRW			0.03
<3,000,000	103 (23.6)	26 (17.5)	
3,000,000–<5,000,000	190 (43.5)	56 (37.6)	
≥5,000,000	144 (33.0)	67 (45.0)	
Education level ^4^			0.2
High school graduation or lower	150 (34.4)	42 (28.2)	
College or university graduation or higher	286 (65.6)	107 (71.8)	
Smoking status			0.1
Non-smoker	266 (60.9)	103 (69.1)	
Former smoker	79 (18.1)	26 (17.5)	
Smoker	92 (21.1)	20 (13.4)	
Alcohol drinking, yes ^5^	346 (79.2)	114 (76.5)	0.5
Body mass index, kg/m^2 6^			0.8
Underweight/normal	230 (52.6)	82 (55.0)	
Overweight	115 (26.3)	35 (23.5)	
Obese	92 (21.1)	32 (21.5)	
Medical condition, yes			
Hypertension	37 (8.5)	12 (8.1)	0.9
Hyperlipidemia	47 (10.8)	11 (7.4)	0.2
Diabetes mellitus	7 (1.6)	0 (0.0)	-
Family history of disease, yes	279 (63.8)	150 (70.5)	0.1
Hypertension	124 (44.4)	50 (47.6)	0.6
Hyperlipidemia	20 (7.2)	10 (9.5)	0.4
Diabetes mellitus	83 (29.8)	34 (32.4)	0.6
Cardiovascular disease	78 (28.0)	31 (29.5)	0.8
Cancer	122 (43.7)	45 (42.9)	0.9

Abbreviation: KRW, Korean Republic Won. ^1^ Data are shown as *n* (%) or mean ± standard deviation. ^2^ VMS includes single vitamin, single mineral, and a vitamin/mineral combination. ^3^ *p* values are derived from the chi-square test for categorical variables, and generalized linear regression analysis for continuous variables. ^4^ These categories had missing data, and the total *n* values are not identical. ^5^ Alcohol drinking was assessed by inquiring about the typical frequency of alcohol consumption over the past year. ^6^ Underweight/normal, <23 kg/m^2^, overweight, 23–<25 kg/m^2^, obese, ≥25 kg/m^2^ based on BMI criteria from the World Health Organization for Asian populations [31].
